# Bacteriological Profile and Drug Resistance Patterns of Blood Culture Isolates in a Tertiary Care Nephrourology Teaching Institute

**DOI:** 10.1155/2014/153747

**Published:** 2014-04-07

**Authors:** Kalpesh Gohel, Amit Jojera, Shailesh Soni, Sishir Gang, Ravindra Sabnis, Mahesh Desai

**Affiliations:** Muljibhai Patel Urological Hospital and Society for Research in Nephro-Urology, Dr. V. V. Desai Road, Nadiad, Gujarat 387001, India

## Abstract

Blood stream infections can lead to life threatening sepsis and require rapid antimicrobial treatment. The organisms implicated in these infections vary with the geographical alteration. Infections caused by MDR organisms are more likely to increase the risk of death in these patients. The present study was aimed to study the profile of organisms causing bacteremia and understand antibiotic resistance patterns in our hospital. 1440 blood samples collected over a year from clinically suspected cases of bacteremia were studied. The isolates were identified by standard biochemical tests and antimicrobial resistance patterns were determined by CLSI guidelines. Positive blood cultures were obtained in 9.2% of cases of which Gram-positive bacteria accounted for 58.3% of cases with staph aureus predominance; gram negative bacteria accounted for 40.2% with enterobactereciea predominence; and 1.5% were fungal isolates. The most sensitive drugs for Gram-positive isolates were vancomycin, teicoplanin, daptomycin, linezolid, and tigecycline and for Gram-negative were carbapenems, colistin, aminoglycosides, and tigecycline. The prevalence of MRSA and vancomycin resistance was 70.6% and 21.6%, respectively. ESBL prevalence was 39.6%. Overall low positive rates of blood culture were observed.

## 1. Introduction

Blood stream infections range from self-limiting infections to life threatening sepsis that requires rapid and aggressive antimicrobial treatment [1]. A wide spectrum of organisms has been described that cause blood stream infections and this spectrum is subject to geographical alteration [2–5]. Increasing antimicrobial resistance is a worldwide concern. The prevalence of resistance of blood borne isolates is increasing and it also varies in accordance with geographical and regional location. The infection caused by MDR organisms is more likely to prolong the hospital stay, increase the risk of death, and require treatment with more expensive antibiotics. In almost all cases, antimicrobial therapy is initiated empirically before the results of blood culture are available. Keeping in mind the high mortality and morbidity associated with septicemia, right choice of empiric therapy is of importance [6]. Therefore, the present study was undertaken to analyze the various organisms causing septicemia and their antibiotic resistance patterns, as it would be a useful guide for clinicians initiating the empiric antibiotic therapy.

## 2. Materials and Methods

A total of 1440 samples from clinically suspected cases of bacteremia were studied at Muljibhai Patel Urological Hospital for a period of one year from October 2012 to September 2013. Our institute is 140-bedded teaching hospital which caters to all kinds of nephrology and urology patients including moderate size of hemodialysis programme as well as kidney transplant programme. All the samples were collected from indoor patients in our hospital during the study period and processed in the central laboratory.

Blood was collected from 2 different sites (avg. 8 mL per site) 20 minutes apart in every patient using strict aseptic precautions and inoculated immediately into BacT/ALERT FA plus—aerobic blood culture bottles with 0.025% of sodium polyanethol sulfonate as anticoagulant. In pediatric cases 1-2 mL of blood was inoculated in BacT/ALERT PF plus pediatric blood culture bottles. After collection these bottles were immediately incubated in BacT/ALERT 3D (manufactured by bioMerieoux)—a fully automated blood culture system for detection of growth in blood culture. The negative results were followed up to 7 days and final report was issued. While, in case of a positive growth, the BacT/ALERT automatically gives an alert. The positive bottles were then subcultured on chrome agar. From the colonies on chrome agar 0.5 McFarland suspension was prepared which was then subjected to identification and susceptibility testing on Mini API (*n* = 50) till February 2013 or Vitek 2 (*n* = 82) from March 2013 onwards (manufactured by bioMerieoux)—which is a fully automated system for identification of organism and antimicrobial susceptibility testing as per the CLSI 2013 guidelines. The ESBL status was determined by Mini API/Vitek 2 as per the CLSI guidelines and was not subjected to any further testing.

## 3. Results

During the study period, 1440 blood cultures were analyzed of which 132 microorganisms were isolated, out of which 130 were bacterial isolates and 2 were fungal isolate, that is,* Candida albicans*. Their mean age was 48.6 ± 14.8 years of which 89 were males and 43 were females. During study period we did not observe multiple positive blood cultures from any patient. The distribution and percentage of various bacterial and fungal isolates are shown in Table 1.

Among the Gram-positive isolates, the predominant isolate was* Staphylococcus aureus* as shown in Table 2 which exhibited least resistance to tetracycline, doxycycline, vancomycin, teicoplanin, tigecycline, daptomycin, and linezolid. Oxacillin resistance (MRSA) was 70.6% in these strains. Vancomycin resistance in* Staphylococcus aureus* isolates was 21.6%. The vancomycin intermediate* Staphylococcus aureus* was observed in 1 patient. In VRSA strains the MIC for vancomycin was ≥32. This was not confirmed further by reference MIC testing.

Other Gram-positive isolates coagulase negative staphylococcal strains (CONS) showed least resistance to gentamicin, quinolones, co-trimoxazole, tigecycline, linezolid, and tetracycline. Kocuria rosea showed no resistance to tetracycline and co-trimoxazole while* Micrococcus* showed least resistance to tetracycline, vancomycin, tigecycline, and levofloxacin. Enterococci showed least resistance to tetracycline, teicoplanin, and tigecycline.* Streptococcus* was isolated in one case only.

Among the Gram-negative isolates, the predominant isolates were* E. coli* and* Klebsiella* in 33 of 53 (62.3%) as highlighted in Table 3 of which 21 (39.6%) were ESBL producers.* E. coli* isolates showed least resistance to carbapenems, aminoglycosides, and tigecycline and moderate resistance to beta-lactam beta-lactamase inhibitors.* Klebsiella* showed least resistance to carbapenems and moderate resistance to aminoglycosides, tigecycline, and beta-lactam beta-lactamase inhibitor combination.* Pseudomonas* showed least resistance to carbapenems, piperacillin-tazobactam, and aminoglycosides. Other Gram-negative isolates were* Burkholderia* in 6 cultures,* Sphingomonas* in 4,* Acinetobacter* in 2, and* Moraxella* in 1 culture. Eight isolates including 2 CONS, 2* Micrococcus*, 2* Sphingomonas*,* 1 Burkholderia*, and 1* Moraxella* were considered contaminated based on clinical and supporting laboratory indicators.

## 4. Discussion

In the present paper, blood culture positivity was seen in 132 of 1440 (9.2%) cases which is quite similar to Mehta et al. [7] and China and Gupta [8] but quite lower to other studies of Kamga et al. [9], Kavitha et al. [10], and Roy et al. [11]. We feel the low incidence in our paper is due to various reasons. Majority of the patients reported to us are referred by other specialists or hospitals and these patients were offered antibiotics elsewhere before they reached our hospital. Many patients developed infections after hospitalization or after surgery by which they already had been given antibiotics before sampling of blood for culture.

The incidence of Gram-positive organisms was 77/132 (58.3%) while 53/132 (40.2%) were Gram-negative isolates in our paper. It is in accordance with the studies of China and Gupta [8], Kamga et al. [9], Anbumani et al. [12], and Karlowsky et al. [13] who reported similar incidencesbut in most of the studies like Mehta et al. [7], Mehdinejad et al. [14], Barati et al. [15], and Ayobola et al. [16] Gram-negative organisms have taken over Gram-positive organisms in hospital settings. This difference could be related to an active dialysis programme and substantial contribution of dialysis line or catheter related infections which are usually of Gram-positive nature. This also indicates that infections by Gram-positive organisms constitute a significant threat to septicemia in our locale and the spectrum of organisms is subject to geographical alterations.

*Staphylococcus* was isolated in 38.6% (*n* = 51) of cases and CONS in 4.5% of cases in the present paper. The isolation of* Staphylococcus aureus* is consistent with the study of Arora and Devi [17], Roy et al. [11], and Karlowsky et al. [13] where the reported isolation of the organism was 27.3%, 14%, and 16.5%, respectively. However, reported isolation of CONS was 20.16%, 16.5%, and 42%, respectively, in these studies which is quite higher than isolation of CONS seen in our study but in accordance with Anbumani et al. [12] where* Staphylococcus aureus* is reported as 36.4% and CONS as 1.12%. Given that CONS isolated from blood are often skin contaminants which are clinically insignificant [1–5], we suspect that the observed low isolation of CONS in our paper could be due to or related to strict aseptic practices of collection method followed for blood sampling of blood culture. The burden of other Gram-positive isolates was much lesser than* Staphylococcus aureus* which is in accordance with these studies.

*Enterococcus* was isolated in 3.8% (*n* = 5) of cases. Out of these 4 were* Enterococcus faecalis* and 1 was* Enterococcus gallinarum*. Amongst the 4* Enterococcus faecalis* 2 were vancomycin sensitive and 2 were vancomycin resistant, while* Enterococcus gallinarum* was moderately sensitive to vancomycin.

*E. coli* and* Klebsiella* (25%) were the predominant Gram-negative isolates in our paper which is in accordance with other studies of Mehta et al. [7], Karlowsky et al. [13], Kamga et al. [9], and China and Gupta [8]. We also observed similar frequency of* Pseudomonas* and* Acinetobacter* as in these studies but we did not observe any isolate of* Salmonella* which is isolated in the frequency of 10 to 20% in these studies. Generally,* Salmonella* is community-acquired infection in general population which gains entry via feco-oral route. Since we cater to specific renal population, that might possibly be the causative factor for this observed difference of* Salmonella*.

We also observed that significant proportion of our patient pool is immunocompromised due to CKD status or postkidney transplantation status which led to bacteremia with various organisms like* Burkholderia*,* Sphingomonas*,* Moraxella*,* Kocuria*, and* Micrococcus* which commonly does not lead to bacteremia in healthy nonimmunocompromised individuals.

*Staphylococcus aureus* isolates in our study exhibited oxacillin resistance of 70.6% which is quite high from the studies of Kamga et al. [9], Kavitha et al. [10], China and Gupta, [8] and Karlowsky et al. [13] who reported a percentage of 18%, 40.8%, 49.5%, and 29%. This is in accordance with the studies of Garg et al. [6] who reported a percentage of 75.6%. Vancomycin resistance in our* Staphylococcus* isolates was 21.6% which is in accordance with Kamga et al. [9] who recorded an isolation of 32%. But it is in contrast to the studies of Karlowsky et al. [13], China and Gupta [8], Garg et al. [6], Kavitha et al. [10], and Roy et al. [11] who reported no resistance to vancomycin. The increasing glycopeptide resistance in our study could be due to widespread usage of the drug in the empirical treatment protocol of suspected CRBSI in dialysis population. However, the current resistance pattern emphasizes the importance of strict antibiotic policy to prevent emergence and spread of antibiotic resistance.

In view of significant oxacillin and vancomycin resistance of 70.6% and 21.6%, respectively, in staphylococcal isolates, drugs like clindamycin, linezolid, daptomycin, and teicoplanin should be considered in the treatment of MRSA before vancomycin. Vancomycin resistant* Enterococcus* (VRE) in our study is 40% (2/5) which is in accordance with the studies like Garg et al. [6] and Karlowsky et al. [13] who reported 16.6% and 35.8%, respectively.

Among the Gram-negative isolates, the Enterobacteriaceae isolates in our study showed very poor sensitivity to quinolones, penicillins, and cephalosporins. However combining BL+BLI did improve the sensitivity. Least resistance was observed with carbapenems, colistin, aminoglycosides, and tigecycline. We could not compare the sensitivity pattern of carbapenems, colistin, and tigecycline with other studies as these drugs were not tested in majority of the other studies.

ESBL producers detected in our study were 39.6% which is in accordance with the study of Kavitha et al. [10] and Arora and Devi [17] who reported prevalence of ESBL producers as 32% and 34.4%, respectively.

## 5. Conclusion

*Staphylococcus aureus* and organisms belonging to Enterobacteriaceae family are the leading causes of septicemia. The most sensitive drugs for Gram-positive isolates were tetracycline, teicoplanin, vancomycin, clindamycin, daptomycin, and linezolid and the most sensitive drugs for Gram-negative bacteria were carbapenems, colistin, aminoglycosides, and tigecycline. Clinicians should exercise caution in their use of vancomycin in order to preserve this useful antibiotic and prolong its therapeutic usefulness and replace its use by drugs like teicoplanin, daptomycin, and linezolid. Increasing incidence of drug resistant organisms like MRSA, VRE, and ESBL producers raises serious concerns about antibiotic resistance and mandates strict antibiotic policy on a large scale. As the practice of prescribing antibiotics is completely unregulated, cheap generics are available, usage of all kinds of antibiotics for even minor illnesses is widespread, and there are not many newer antimicrobials in research pipeline, it is foreseen that if the same kind of practice and scenario continues the antibiotic resistance is likely to go up and we will face serious crisis of antibiotics in near future.

## Figures and Tables

**Table 1:** Distribution of isolates in blood cultures.

Type	Numbers	Percentage
*Staphylococcus *	51	38.6
*E. coli *	20	15.2
*Klebsiella *	13	9.8
*Pseudomonas *	7	5.3
*Kocuria *	7	5.3
*Micrococcus *	7	5.3
*Burkholderia *	6	4.5
Coagulase-negative staphylococci	6	4.5
*Enterococcus *	5	3.8
*Sphingomonas *	4	3.0
*Candida *	2	1.5
*Acinetobacter *	2	1.5
*Moraxella *	1	0.8
*Streptococcus *	1	0.8

**Table 2:** Drug resistance pattern of major Gram-positive isolates.

Antimicrobial tested	*Enterococcus* (*n* = 5)		*Staphylococcus aureus* (*n* = 51)	
	Mini API (*n* = 3)	Vitek (*n* = 2)	Mini API (*n* = 21)	Vitek (*n* = 30)
Amoxicillin	NP	NP	100	100
Cefotaxime + clavulanate	NP	50	NP	37
Ceftazidime + clavulanate	NP	50	NP	37
Cefepime + tazobactam	NP	50	NP	30
Tigecycline	NP	0	NP	NP
Gentamycin	33	50	NP	NP
Amikacin	33	50	NP	NP
Ciproflox	100	100	90	87
Levoflox	100	100	75	80
Nalidixic acid	100	100	NP	NP
Nitrofurantoin	100	100	0	78
Tetracycline	33	50	10	27
Doxycycline	33	50	10	27
Minocycline	33	50	10	27
Oxacillin	NP	NP	73	67
Vancomycin**	50	50	20	22
Teicoplanin	33	50	NP	0
Daptomycin	NP	0	NP	6
Linezolid	NP	0	NP	3

NP: drug not in panel; **see Section 4.

Figures in the table are expressed in percentages.

**Table 3:** Drug resistance pattern of major Gram-negative isolates.

Antimicrobial tested	*E. Coli* (*n* = 20)		*Klebsiella* (*n* = 13)^##^	*Pseudomonas* (*n* = 7)	
	Mini API (*n* = 12)	Vitek (*n* = 8)	Vitek (*n* = 13)	Mini API (*n* = 1)	Vitek (*n* = 6)
Amoxicillin	92	100	100	100	100
Ceftriaxone	92	100	100	100	100
Ceftazidime	92	87.50	92	0	67
Cefepime	84	75	92	0	50
Amoxicillin + clavulanate	84	100	100	100	100
Cefotaxime + clavulanate	40	37.50	72	100	83
Ceftazidime + clavulanate	40	37.50	54	100	67
Ticarcillin + clavulanate	58	NP	NP	0	NP
Piperacillin + tazobactam	50	50	77	0	33
Cefoperazone + sulbactam	NP	12.50	62	NP	33
Cefepime + tazobactam	NP	37.50	54	NP	33
Imipenem	10	0	54	0	33
Meropenem	10	12.50	70	0	33
Ertapenem	NP	NP	62	NP	NP
Colistin	NP	0	8	0	0
Tigecycline	NP	0	54	NP	67
Gentamycin	42	37.50	54	0	33
Amikacin	30	12.50	46	0	33
Ciproflox	92	87.50	77	0	33
Levoflox	92	87.50	77	0	33
Nalidixic acid	92	87.50	84	NP	NP
Nitrofurantoin	58	70	84	100	83
Tetracycline	75	75	84	100	83
Doxycycline	75	75	84	100	83
Minocycline	75	75	84	100	83

Figures are in percentage; N.P: drug not in panel; ^##^all the *Klebsiella* isolates were tested on Vitek 2.
